# Microbial metabolic memory in inflammatory bowel disease: microbiota-derived metabolites, host–microbe reprogramming, and relapse susceptibility

**DOI:** 10.3389/fmicb.2026.1893807

**Published:** 2026-07-29

**Authors:** Xiaozhen Cheng, Tao Zhang, Mengying Zhu, Zhengchao Pan, Meng Chen, Weilin Li, Ru Man, Xiangdong Zhao, Yongduo Yu

**Affiliations:** 1 Shenzhen Traditional Chinese Medicine Hospital, Shenzhen, China; 2 Liaoning University of Traditional Chinese Medicine, Shenyang, China; 3 Second Affiliated Hospital, Liaoning University of Traditional Chinese Medicine, Shenyang, China

**Keywords:** gut microbiota, host–microbe interaction, inflammatory bowel disease, metabolic memory, metabolomics, microbial metabolites, mucosal immunity, relapse

## Abstract

Inflammatory bowel disease (IBD) is increasingly recognized as a disorder of disrupted host–microbe metabolic communication rather than a consequence of microbial dysbiosis alone. Gut microbiota-derived metabolites, including short-chain fatty acids, tryptophan derivatives, bile acid metabolites, polyamines, lactate, succinate, and trimethylamine N-oxide, act as functional mediators linking microbial ecological changes to epithelial barrier integrity, mucosal immune activation, inflammatory amplification, and tissue repair. However, most existing discussions have focused on individual metabolites or isolated immune pathways, leaving the dynamic and disease-stage-specific nature of microbial metabolic remodeling insufficiently defined. In this review, we reframe IBD pathogenesis from the perspective of microbial metabolic memory, emphasizing how persistent alterations in microbial metabolic output may imprint epithelial and immune cell responses even after clinical remission. We summarize how protective metabolite depletion and proinflammatory metabolite accumulation contribute to immune tolerance breakdown, barrier dysfunction, inflammatory propagation, extraintestinal immune manifestations, and relapse susceptibility. We further discuss how mucosal inflammation reciprocally reshapes microbial niches, oxygen tension, nutrient availability, and metabolic pathway activity, thereby establishing self-reinforcing host–microbe feedback loops. Finally, we evaluate emerging therapeutic strategies, including metabolite supplementation, blockade of proinflammatory metabolic signaling, microbiota-based metabolic remodeling, engineered probiotics, and metabolomics-guided precision interventions. This metabolic-memory framework may provide a more integrated basis for understanding IBD recurrence and for developing microbiota-targeted strategies aimed at restoring durable intestinal homeostasis.

## 1 Introduction

Inflammatory bowel disease (IBD), mainly comprising Crohn’s disease (CD) and ulcerative colitis (UC), is a group of chronic relapsing inflammatory disorders characterized by complex and multifactorial pathogenesis. Its development involves intricate interactions among genetic susceptibility, environmental exposures, gut microbiota dysbiosis, and mucosal immune dysregulation (Hashash et al., 2025; Hu S. et al., 2024). Although advances in anti-inflammatory therapies and biologic agents have substantially improved clinical outcomes in a proportion of patients, disease relapse remains common, suggesting that unresolved biological alterations may persist even during clinical remission.

With the rapid development of metagenomics, metabolomics, and integrated multi-omics approaches, increasing attention has been directed toward the role of gut microbiota and microbial-derived metabolites in IBD pathogenesis. Accumulating evidence indicates that IBD is accompanied not only by alterations in microbial composition but also by extensive remodeling of microbial metabolic profiles. These metabolic disturbances may represent not merely secondary consequences of dysbiosis, but also potential mediators involved in shaping host immune responses and inflammatory states (Poppe et al., 2024).

Gut microorganisms produce a wide range of bioactive metabolites, including short-chain fatty acids (SCFAs), secondary bile acids, tryptophan-derived metabolites, polyamines, as well as lactate, succinate, and ATP-related metabolites (Ma et al., 2022). These microbial-derived molecules can influence intestinal epithelial cells and mucosal immune populations through receptor-mediated signaling, metabolic reprogramming, and epigenetic regulation, thereby contributing to the maintenance of barrier integrity and immune homeostasis (Krause et al., 2024; Wu H. et al., 2024). Conversely, mucosal immune responses can also reshape microbial composition and metabolic output through cytokine networks, antimicrobial peptides, and alterations in local microenvironmental conditions, such as oxygen availability and pH, forming a dynamic bidirectional interaction network (Liu et al., 2026a; Neuendorff et al., 2026). When this network becomes disrupted, abnormal metabolic signals may participate in sustaining inflammatory responses and contribute to the chronic inflammatory characteristics of IBD (Michaudel et al., 2023).

Against this background, this review provides an integrated perspective on the bidirectional crosstalk between microbial metabolites and mucosal immunity, summarizing the major mechanisms through which microbial-derived metabolites may regulate immune homeostasis and highlighting their dynamic alterations across different disease stages. Unlike previous reviews primarily organized according to “metabolite categories and signaling pathways,” this review adopts a disease-stage dynamic perspective as the central framework to better illustrate the continuous evolution of metabolic–immune interactions from disease initiation and progression to remission and relapse susceptibility.

Furthermore, to provide a conceptual framework for understanding the persistence of relapse susceptibility in IBD, we introduce the concept of “microbial metabolic memory.” This concept is proposed as an emerging explanatory framework rather than an established pathogenic mechanism and can be operationally considered at two interconnected levels: (i) passive metabolic memory, referring to the persistent presence of low-grade abnormal microbial metabolite profiles during clinical remission due to incomplete restoration of gut microbial ecological homeostasis; and (ii) active metabolic memory, referring to potential long-term functional reprogramming of intestinal immune and epithelial cells induced by abnormal metabolites through epigenetic mechanisms, including histone deacetylase inhibition, DNA methylation, and histone modifications, thereby potentially altering immune response thresholds and maintaining inflammatory susceptibility.

**FIGURE 1:**
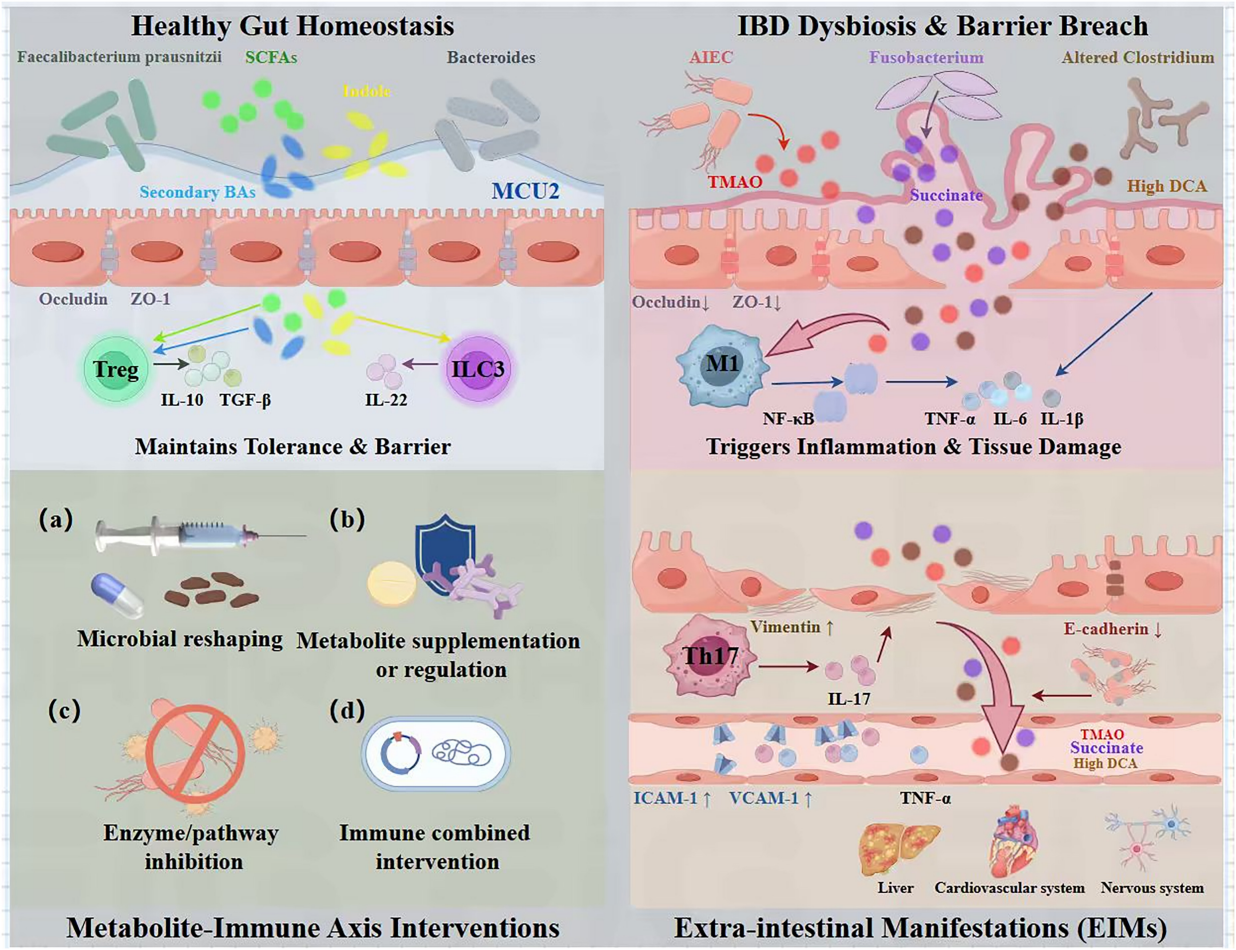
Bidirectional crosstalk between microbial metabolites and mucosal immunity in IBD. Microbial metabolites derived from the gut microbiota, including short-chain fatty acids, bile acid metabolites, tryptophan-derived metabolites, polyamines, and other microbiota-associated metabolites, regulate epithelial barrier integrity, innate immune activation, adaptive immune differentiation, and inflammatory responses through receptor-mediated signaling, immunometabolic reprogramming, and epigenetic regulation. Conversely, mucosal immune responses reshape microbial composition and metabolic activity through cytokines, antimicrobial peptides, secretory IgA, and alterations in the intestinal microenvironment. Disruption of this bidirectional metabolite–immune network contributes to chronic inflammation, tissue injury, fibrosis, and relapse during IBD progression.

Importantly, microbial metabolic memory differs conceptually from related theories, including trained immunity, mucosal immune imprinting, and persistent dysbiosis. While trained immunity mainly describes enhanced memory-like responses of innate immune cells after initial stimulation, mucosal immune imprinting emphasizes long-lasting alterations within local immune environments, and persistent dysbiosis primarily reflects incomplete recovery of microbial ecological structure. In contrast, microbial metabolic memory focuses on microbial metabolites as potential mediators linking ecological alterations of the microbiota with long-term host immune adaptation. Based on this framework, we further integrate the stage-specific characteristics, potential feedback loops, and translational strategies of metabolic–immune interactions in IBD, aiming to provide a conceptual basis for understanding relapse susceptibility and developing metabolite-targeted interventions. The overall framework illustrating the bidirectional crosstalk between microbial metabolites and mucosal immunity in IBD is summarized in Figure 1.

## 2 Microbial metabolite-related alterations in the gut microecology and mucosal immune homeostasis

With the rapid advancement of metagenomics, metabolomics, and integrated multi-omics approaches, alterations in the gut ecosystem associated with IBD have been increasingly characterized. Compared with healthy individuals, patients with IBD commonly exhibit reduced microbial diversity, community restructuring, depletion of beneficial taxa including short-chain fatty acid (SCFA)-producing bacteria, and expansion of potentially pathogenic microorganisms (Kaden et al., 2025; Ning et al., 2023). However, the reported microbial alterations are not always consistent across studies, which may be attributable to differences in disease subtype, anatomical location, disease activity, dietary patterns, medication exposure, and analytical methodologies. Therefore, taxonomic alterations alone may not fully explain the functional consequences of microbiota disruption in IBD, and increasing attention has shifted toward understanding microbiota dysfunction from the perspective of metabolic capacity and functional output (Taubenheim et al., 2025).

At the functional level, microbial compositional changes in IBD are frequently accompanied by extensive alterations in metabolite profiles. Reduced SCFA production capacity, disturbed tryptophan metabolism, impaired bile acid transformation, and alterations in repair-associated metabolites such as polyamines have been reported to be associated with intestinal barrier dysfunction and local immune imbalance (Attema and Kuipers, 2025; Hazime et al., 2025). Compared with individual microbial abundance changes, metabolite profiles may more directly reflect the functional state of microbiota–host interactions. SCFAs are involved in epithelial energy metabolism, tight junction maintenance, and immune tolerance regulation (Takeuchi et al., 2024); tryptophan-derived metabolites participate in epithelial defense and mucosal repair processes (Wang X. et al., 2026); and bile acid metabolites may influence intestinal permeability and immune response thresholds through host receptor-mediated pathways (Paik et al., 2022). Thus, metabolic dysfunction represents an important functional dimension of microbiota disruption in IBD beyond alterations in microbial composition alone.

In addition to metabolic alterations, the mucosal immune environment in IBD exhibits substantial dysregulation. Under physiological conditions, intestinal immunity maintains tolerance toward commensal microorganisms while preserving effective defense against pathogens, a balance supported by coordinated interactions among epithelial barriers, mucus layers, antimicrobial peptides, and innate and adaptive immune responses (Villablanca et al., 2022; Yue et al., 2024). In IBD, impaired barrier integrity, abnormal immune activation, and disruption of the Treg/Th17 balance are associated with altered mucosal immune homeostasis (Han et al., 2026). Microbial-derived metabolites may influence immune cell activation states and cytokine production, thereby participating in immune microenvironment remodeling (Dalal et al., 2025; Yang and Cong, 2021). Conversely, immune responses can reshape microbial composition and metabolic activity through antimicrobial peptides, IgA-mediated microbial selection, and alterations in local oxygen availability (Hu et al., 2026). Therefore, metabolic alterations and mucosal immune dysregulation in IBD are likely interconnected through a dynamic bidirectional regulatory network.

Genetic susceptibility may also contribute to the heterogeneity of metabolic–immune interactions in IBD by modifying host responses to microbial-derived signals. Several key IBD susceptibility genes, including NOD2, ATG16L1, and IL23R, may influence microbial recognition, autophagy processes, and inflammatory signaling pathways, thereby potentially altering host sensitivity and responses to microbial metabolites. For example, impaired NOD2 function may affect epithelial sensing of microbial-associated molecular patterns and modify downstream inflammatory thresholds (Okai et al., 2022). Similarly, ATG16L1-related autophagy defects may influence microbial handling and cellular responses to microbial-derived signals (Gao P. et al., 2022), whereas IL23R-associated pathways may regulate Th17-related immune responses involved in inflammatory amplification (Krause-Kyora et al., 2025). Thus, genetic background may not only influence IBD susceptibility but may also modify the intensity and direction of microbiota–metabolite–immune interactions.

Importantly, current evidence remains insufficient to determine the precise causal direction between metabolic alterations and immune dysregulation in IBD. On one hand, microbial metabolic abnormalities may be associated with impaired barrier function and immune imbalance; on the other hand, persistent inflammation and altered tissue microenvironments may reciprocally reshape microbial composition and metabolic activity. Therefore, metabolic abnormalities observed in IBD may represent both potential contributors to disease progression and secondary consequences of inflammatory states. Future longitudinal studies integrating multi-omics approaches are required to further clarify the temporal relationships and causal mechanisms underlying these interactions.

Overall, alterations in microbial composition and metabolic function establish the foundational ecological state of IBD-associated dysbiosis and provide the biological context for subsequent stage-specific remodeling of metabolic–immune interactions during disease evolution. These alterations may represent an important transition point linking molecular-level disturbances to dynamic pathological changes across different phases of IBD.

## 3 Molecular mechanisms underlying the interaction between microbial metabolites and mucosal immunity

**FIGURE 2:**
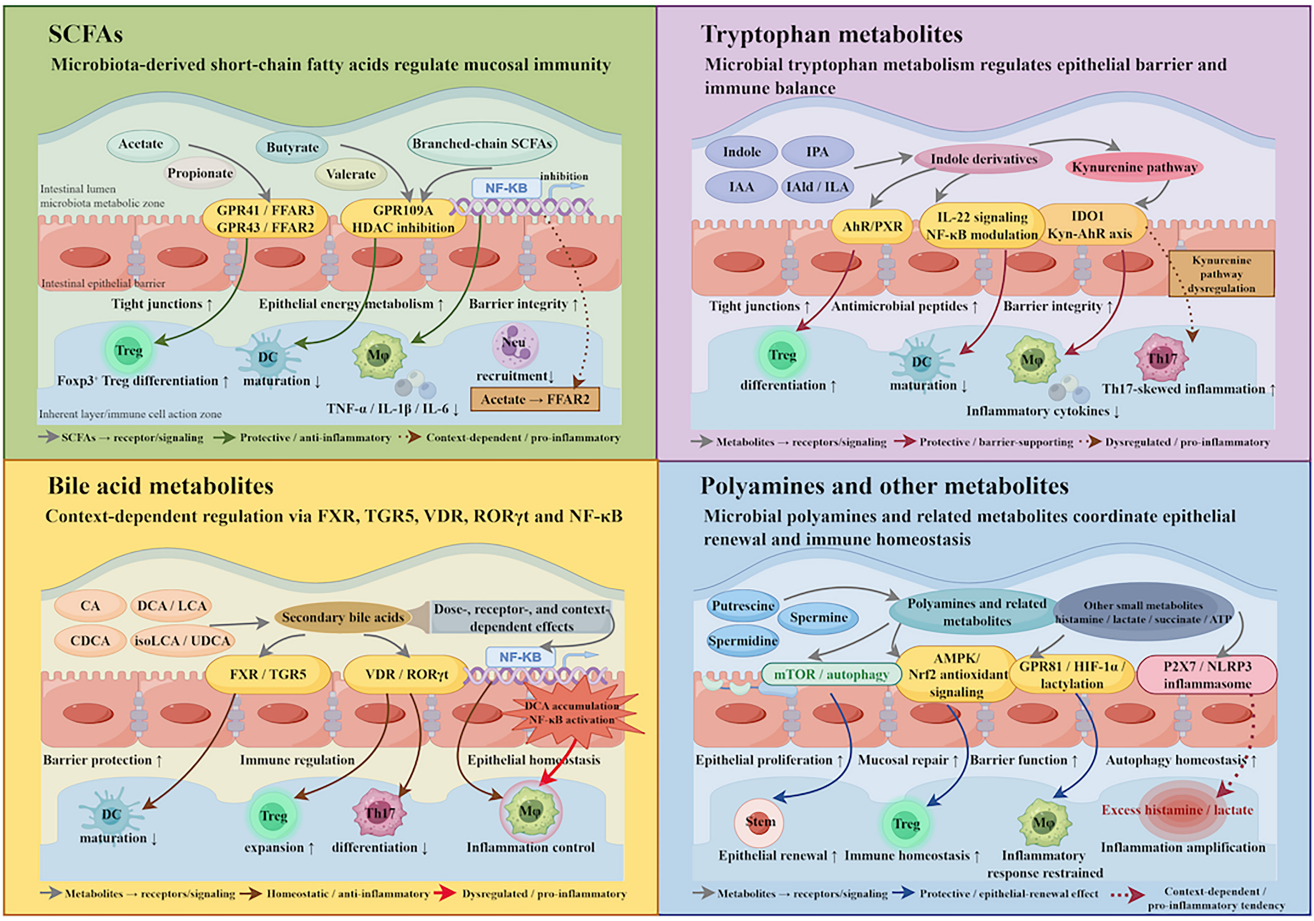
Molecular mechanisms by which microbial metabolites regulate mucosal immunity in IBD. Different microbial metabolites modulate mucosal immunity through distinct molecular pathways. Short-chain fatty acids regulate epithelial barrier integrity and immune tolerance through FFARs and HDAC inhibition. Tryptophan-derived metabolites activate AHR-related signaling and promote IL-22-mediated mucosal defense. Bile acid metabolites regulate Treg/Th17 balance and inflammatory signaling through FXR, TGR5, VDR, and RORγt-associated pathways. Polyamines and other microbiota-associated metabolites further influence epithelial repair, macrophage polarization, inflammasome activation, oxidative stress, and immunometabolic reprogramming. These pathways collectively shape intestinal immune homeostasis and inflammatory responses during IBD.

Microbial-derived metabolites represent important molecular mediators linking microbial functional activity with host mucosal immune responses. Through receptor-mediated sensing, intracellular signaling regulation, immunometabolic modulation, and epigenetic mechanisms, these metabolites can influence epithelial barrier integrity, immune cell activation, and inflammatory responses (Vich Vila et al., 2024). In IBD, alterations in microbial metabolite profiles are closely associated with mucosal immune dysregulation, potentially affecting immune tolerance, barrier homeostasis, and inflammatory responses depending on the specific metabolite, cellular context, and local microenvironment (Liu et al., 2026b; Wehkamp et al., 2026). Conversely, mucosal immune responses can also reshape microbial metabolic activity through cytokine networks, antimicrobial peptides, IgA-mediated microbial selection, and alterations in local ecological conditions (Pabst and Izcue, 2022). The major mechanisms through which microbial metabolites regulate mucosal immunity are summarized in Figure 2.

### 3.1 Regulation of mucosal immunity by microbial metabolites

Gut microbiota-derived metabolites interact extensively with mucosal immune components and may contribute to the initiation, maintenance, and recurrence susceptibility of IBD. Major metabolite classes, including short-chain fatty acids (SCFAs), tryptophan-derived metabolites, bile acid derivatives, polyamines, as well as lactate, succinate, and ATP-related metabolites, can act on intestinal epithelial cells and lamina propria immune populations through receptor signaling, metabolic regulation, and epigenetic modulation (Table 1).

**TABLE 1:** Major target cells, signaling pathways, and immune effects of microbial metabolites in mucosal immunity.

Metabolite	Major target cells/ immune components	Core pathways/ mechanisms	Main immune effects	References
SCFAs				
Acetate	Intestinal epithelial cells	FFAR2/GPR43; energy metabolism regulation	Maintains epithelial metabolism and barrier stability	Hu L. et al., 2024; Pan et al., 2024; Song et al., 2024
Acetate	Neutrophils/Th17 cells	FFAR2-dependent chemotaxis regulation	Regulates inflammatory cell recruitment	Brennan et al., 2021; Huang et al., 2026
Propionate	Treg cells	HDAC inhibition; Foxp3 expression	Enhances immune tolerance	Du et al., 2022; Kang et al., 2021
Butyrate	Intestinal epithelial cells	Energy substrate; tight junction regulation	Enhances barrier integrity	Mavrogeni et al., 2022; Shi et al., 2025
Butyrate	Treg cells	HDAC inhibition; Foxp3-region acetylation	Promotes Treg differentiation and stability	Rangan and Mondino, 2022; Yang W. et al., 2026
Butyrate	DCs	Inhibition of excessive DC maturation	Promotes tolerogenic antigen presentation	Andrusaite et al., 2024; Wu et al., 2023
Butyrate	Macrophages	NF-κB inhibition; M1/M2-like polarization regulation	Reduces pro-inflammatory cytokine release	Niu et al., 2026; Wang W. et al., 2024
Valerate	Treg cells	HDAC inhibition; Foxp3 expression	Promotes Treg differentiation	Chen et al., 2023; Lin et al., 2021
Isobutyrate/isovalerate	Treg cells/macrophages/neutrophils	FFAR2/FFAR3 signaling	Regulates inflammation and barrier repair	Guo et al., 2025; Xing et al., 2025
Tryptophan metabolites				
Indole	Intestinal epithelial cells	AHR-related barrier signaling	Enhances epithelial defense	Li M. et al., 2024; Wang X. et al., 2026
Indole-3-propionic acid	Intestinal epithelial cells/macrophages	AHR/PXR; anti-oxidative stress	Protects barrier function and limits inflammation	Luo et al., 2025; Prasad et al., 2026
Indole-3-aldehyde	ILC3s/intestinal epithelial cells	AHR–IL-22 axis	Promotes mucosal defense and repair	Puccetti et al., 2021; Renga et al., 2022
Indole-3-lactic acid	DCs	Cytokine regulation; epigenetic modification	Regulates T-cell activation	Xie L. et al., 2026; Zhang et al., 2023
Kynurenine	T cells/DCs	AHR; immunometabolic regulation	Regulates Treg/Th17 balance	Liu et al., 2024; Lou et al., 2025
Bile acid metabolites				
Cholic acid	Intestinal epithelial cells/macrophages	FXR/TGR5; bile acid homeostasis	Regulates barrier function and inflammatory threshold	Fu et al., 2025; Yi et al., 2026
Chenodeoxycholic acid	Intestinal epithelial cells/immune cells	FXR-related signaling	Regulates epithelial and local immune responses	Lindner et al., 2024; Yang W. et al., 2026
Deoxycholic acid	Epithelial cells/macrophages/neutrophils	FXR/TGR5; oxidative stress	Bidirectionally regulates inflammatory responses	Ni et al., 2025; Pi et al., 2023
Lithocholic acid	DCs/Th17 cells/Treg cells	VDR; RORγt-related pathways	Suppresses Th17 and enhances Treg regulation	Han et al., 2025; Ridlon and Gaskins, 2024
Iso-lithocholic acid	Th17 cells	RORγt transcriptional inhibition	Suppresses Th17 differentiation	Shi et al., 2022; Wu et al., 2026
3β-hydroxydeoxycholic acid	DCs/Treg cells	Reduced DC immunostimulatory function	Promotes Treg expansion	Thomas et al., 2022; Yan et al., 2021
Ursodeoxycholic acid	DCs/macrophages/epithelial cells	NF-κB inhibition; tolerogenic signaling	Reduces inflammation and protects barrier function	Yu Z. et al., 2026
Polyamines				
Putrescine	Intestinal epithelial cells/macrophages	Regulation of cell proliferation and migration	Participates in epithelial renewal and immune regulation	Cairns et al., 2025; Liu et al., 2019
Spermidine	Intestinal epithelial cells/macrophages/T cells	Autophagy regulation; anti-oxidative stress	Promotes repair and limits inflammation	Liu et al., 2025; Niechcial et al., 2023
Spermine	Intestinal epithelial cells/immune cells	Barrier repair; cytokine regulation	Supports mucosal homeostasis	Liu et al., 2022; Nakamura and Matsumoto, 2025
Other metabolites				
Lactate	Macrophages/T cells/epithelial cells	GPR81; HIF-1α; lactylation	Regulates immunometabolism and local inflammation	Feng et al., 2022; Yang et al., 2022
Succinate	Macrophages/DCs/epithelial cells	SUCNR1; HIF-1α stabilization	Enhances IL-1β-related inflammation	Bernabe-Yepes and Zazueta, 2026; Yang et al., 2024
ATP	DCs/macrophages/neutrophils/T cells	P2X7; NLRP3 inflammasome	Promotes IL-1β release and Th17 inflammation	Bockstiegel et al., 2023; Ranéia et al., 2021
Adenosine	T cells/macrophages/DCs	A2A/A2B receptors	Limits excessive immune activation	Cheng T. et al., 2025; Fu et al., 2023
TMAO	Macrophages/vascular endothelial cells	Inflammatory signaling; immunometabolic alteration	Promotes systemic inflammatory activation	Wen et al., 2025; Xiang et al., 2026
Histamine	Mast cells/T cells/epithelial cells	Histamine receptor signaling	Regulates permeability and immune activation	Gao S. et al., 2022; Krempski et al., 2024

Given the diversity of microbial metabolites, their cellular targets, and regulatory mechanisms, the major metabolite–immune interactions are systematically summarized in Table 1 to provide a structured overview and avoid unnecessary repetition within the main text.

Among microbial metabolites, SCFAs represent one of the most extensively studied groups. Through pathways involving G-protein-coupled receptors, including GPR41, GPR43, and GPR109A, as well as histone deacetylase (HDAC) inhibition, SCFAs participate in epithelial energy metabolism, tight junction maintenance, and regulatory T cell (Treg) differentiation. These effects generally support immune tolerance and barrier stability; however, their biological outcomes are highly dependent on cellular context, inflammatory status, and metabolite availability.

Tryptophan-derived metabolites primarily regulate mucosal immunity through the aryl hydrocarbon receptor (AHR) pathway, influencing epithelial barrier function, innate lymphoid cell 3 (ILC3)-associated responses, and interleukin-22 (IL-22)-mediated mucosal defense. In IBD, alterations in tryptophan metabolism, including potential shifts toward the kynurenine pathway, have been associated with immune metabolic remodeling and altered inflammatory responses.

Bile acid metabolites regulate epithelial function and immune response thresholds through multiple host receptors, including the farnesoid X receptor (FXR), Takeda G protein-coupled receptor 5 (TGR5), and vitamin D receptor (VDR). Their biological effects are highly context-dependent and may contribute either to immune homeostasis or inflammatory regulation depending on bile acid composition, concentration, and local inflammatory conditions.

Polyamine metabolites are closely associated with epithelial regeneration, cellular proliferation, and tissue repair processes. Their immunomodulatory effects are also influenced by disease stage and microenvironmental conditions, suggesting that polyamines may function as dynamic regulators rather than uniformly protective molecules.

In addition, metabolites such as lactate, succinate, ATP, and adenosine regulate immune responses through pathways involving GPR81, SUCNR1, P2 × 7 receptors, and adenosine receptors. While some of these metabolites may enhance inflammatory signaling under specific conditions, others may contribute to immune suppression or resolution, further highlighting the complexity and context dependence of microbial metabolite-mediated immune regulation.

Importantly, the biological functions of microbial metabolites are not static but may undergo dynamic remodeling during different stages of IBD according to changes in microbial composition, inflammatory microenvironments, and host immune states. Therefore, the immunological effects of individual metabolites should not be interpreted as intrinsically protective or pro-inflammatory, but rather as outcomes determined by the interaction between metabolite properties and surrounding biological contexts.

Beyond qualitative differences among metabolites, their immunological effects may also exhibit non-linear concentration–response relationships. The same metabolite may induce distinct or even opposing biological outcomes at different concentration ranges. For example, SCFAs at physiological concentrations generally promote Treg differentiation and epithelial barrier maintenance, whereas excessive accumulation or abnormal local concentrations may negatively affect epithelial homeostasis under certain conditions. Similar concentration-dependent effects may also occur with bile acid metabolites and other microbial-derived molecules. These observations indicate that metabolite functions depend not only on molecular identity but also on local concentration gradients and tissue microenvironmental conditions. However, the physiological and pathological concentration ranges of many microbial metabolites within the intestinal ecosystem remain insufficiently defined, representing an important unresolved challenge in the field of metabolic–immune research.

### 3.2 Network interactions among microbial metabolites

Beyond the effects of individual metabolites on mucosal immunity, microbial-derived metabolites do not function as isolated molecules but rather form interconnected metabolic networks through synergistic, antagonistic, and compensatory interactions. Such metabolite–metabolite interactions may collectively shape immune homeostasis, particularly under conditions where multiple metabolic pathways are simultaneously altered in IBD. Therefore, understanding metabolic networks rather than individual metabolites alone may provide a more comprehensive framework for interpreting microbiota–immune dysregulation (Elmassry et al., 2025; Schirmer et al., 2024).

Synergistic interactions among metabolites may occur when different molecules regulate complementary biological processes. For example, butyrate and indole-3-propionic acid (IPA) may exert coordinated effects on intestinal barrier maintenance. Butyrate can enhance epithelial integrity and Treg differentiation through HDAC inhibition and related signaling pathways (Korsten et al., 2023), whereas IPA may promote epithelial defense and barrier repair through activation of AHR-related pathways (Zhao et al., 2025). Their combined actions may contribute to improved mucosal barrier function and immune regulation.

Conversely, metabolites with opposing biological properties may counterbalance each other through antagonistic signaling interactions. For instance, butyrate and trimethylamine N-oxide (TMAO) may exert opposing effects on inflammatory regulation. Butyrate has been associated with reduced NF-κB activation and inflammatory cytokine production (Chen M. et al., 2025; Cheng T. et al., 2025), whereas TMAO may enhance inflammatory signaling and endothelial immune activation under certain conditions (Li W. et al., 2024). The relative balance between these metabolic signals may influence the intensity and persistence of inflammatory responses.

In addition, microbial metabolites may interact indirectly through shared receptors or overlapping signaling pathways. For example, bile acid derivatives and tryptophan-derived metabolites can both regulate immune responses through nuclear receptor-related pathways, including FXR, VDR, and AHR signaling (Zhu et al., 2025). Such interconnected signaling networks suggest that microbial metabolites may function as an integrated regulatory system rather than independent molecular mediators.

### 3.3 Reverse regulation of microbial metabolism by mucosal immunity

Although microbial-derived metabolites actively regulate mucosal immune responses, the immune system can also reshape microbial ecological niches, spatial distribution, and intestinal environmental conditions, thereby influencing microbial metabolic production and transformation (Gao et al., 2025). Under physiological conditions, secretory IgA, antimicrobial peptides, mucus layers, and epithelial barriers collectively restrict excessive microbial attachment and mucosal invasion, contributing to the maintenance of relatively stable microbial localization and metabolic activity (Simpson et al., 2026).

In IBD, impaired barrier function, immune cell infiltration, and persistent inflammatory signaling may alter luminal oxygen availability, nutrient accessibility, mucus composition, and antimicrobial peptide profiles, thereby reshaping microbial community structure and metabolic output (Zong et al., 2024). At the ecological level, secretory IgA can selectively coat specific commensal or potentially pathogenic bacteria, influencing their mucosal localization and ecological fitness (Hockenberry et al., 2023). Antimicrobial peptides produced by Paneth cells and intestinal epithelial cells, including α-defensins and Reg3γ, contribute to the formation of a chemical barrier and may influence microbial colonization patterns (Frazier et al., 2022). Consequently, alterations in IgA coating patterns, antimicrobial peptide expression, or mucus organization may modify the distribution of SCFA-producing bacteria, bile acid-transforming microorganisms, and tryptophan-metabolizing microbial populations (Josephson et al., 2026).

Inflammatory microenvironments may further reshape microbial metabolic capacity through alterations in intestinal redox conditions. During inflammation, epithelial injury and immune activation may increase luminal oxygen availability, resulting in a shift from a relatively anaerobic environment toward a more oxidative state (Hsu et al., 2023). Because many butyrate-producing microorganisms are strict anaerobes, such environmental changes may negatively affect their ecological fitness and metabolic activity, whereas facultative anaerobic bacteria may gain competitive advantages under these conditions (Kroon et al., 2025). In addition, reactive oxygen species, reactive nitrogen species, and inflammatory mediators released by neutrophils and macrophages may influence local redox balance and nutrient availability, potentially affecting SCFA production, bile acid transformation, and tryptophan metabolism (Miller et al., 2023).

Cytokine networks may also contribute to the regulation of microbial metabolic niches. Pro-inflammatory cytokines, including TNF-α, IFN-γ, and IL-17, may influence epithelial barrier integrity, mucus secretion, and antimicrobial peptide expression, thereby modifying microbial colonization environments (Brabec et al., 2023; Yue et al., 2020). IL-22, in contrast, may contribute to barrier maintenance through epithelial repair and antimicrobial peptide induction, although its effects may vary depending on disease stage and inflammatory context. During persistent inflammation, alterations in epithelial metabolic states and mucus glycosylation patterns may modify nutrient availability for microbial utilization, potentially contributing to reduced production of beneficial metabolites and enrichment of inflammation-associated metabolic signals (Chatterjee et al., 2025).

Therapeutic interventions in IBD may further influence microbial metabolism indirectly through modulation of mucosal inflammation and ecological conditions. Corticosteroids, immunosuppressive agents, biologics, antibiotics, and nutritional interventions can alter inflammatory states, microbial composition, and metabolic pathway activity to varying degrees (Xu et al., 2024). Following inflammatory remission, partial restoration of epithelial barrier function and anaerobic conditions may be associated with recovery of beneficial microbial populations and SCFA production capacity (Yang et al., 2025). Conversely, antibiotic exposure or prolonged immunomodulatory treatment may contribute to persistent alterations in microbial structure and metabolic pathways, including bile acid and tryptophan metabolism (Shao-Yu et al., 2025).

Overall, immune regulation of microbial metabolism extends beyond immediate changes in microbial composition and metabolite production. Through continuous modulation of intestinal ecological conditions, redox balance, immune thresholds, and barrier integrity, mucosal immunity may influence the long-term stability and resilience of microbial metabolic networks.

## 4 Interactions between microbial metabolites and mucosal immunity during IBD progression

To bridge molecular mechanisms with disease phenotypes, this section adopts a disease-stage-oriented framework to analyze the dynamic alterations of microbial metabolites and their immune consequences throughout IBD evolution. Rather than focusing solely on individual metabolite functions, we emphasize how dominant metabolic pathways and immune responses are progressively remodeled during disease initiation, inflammatory progression, remission, and relapse susceptibility.

**FIGURE 3:**
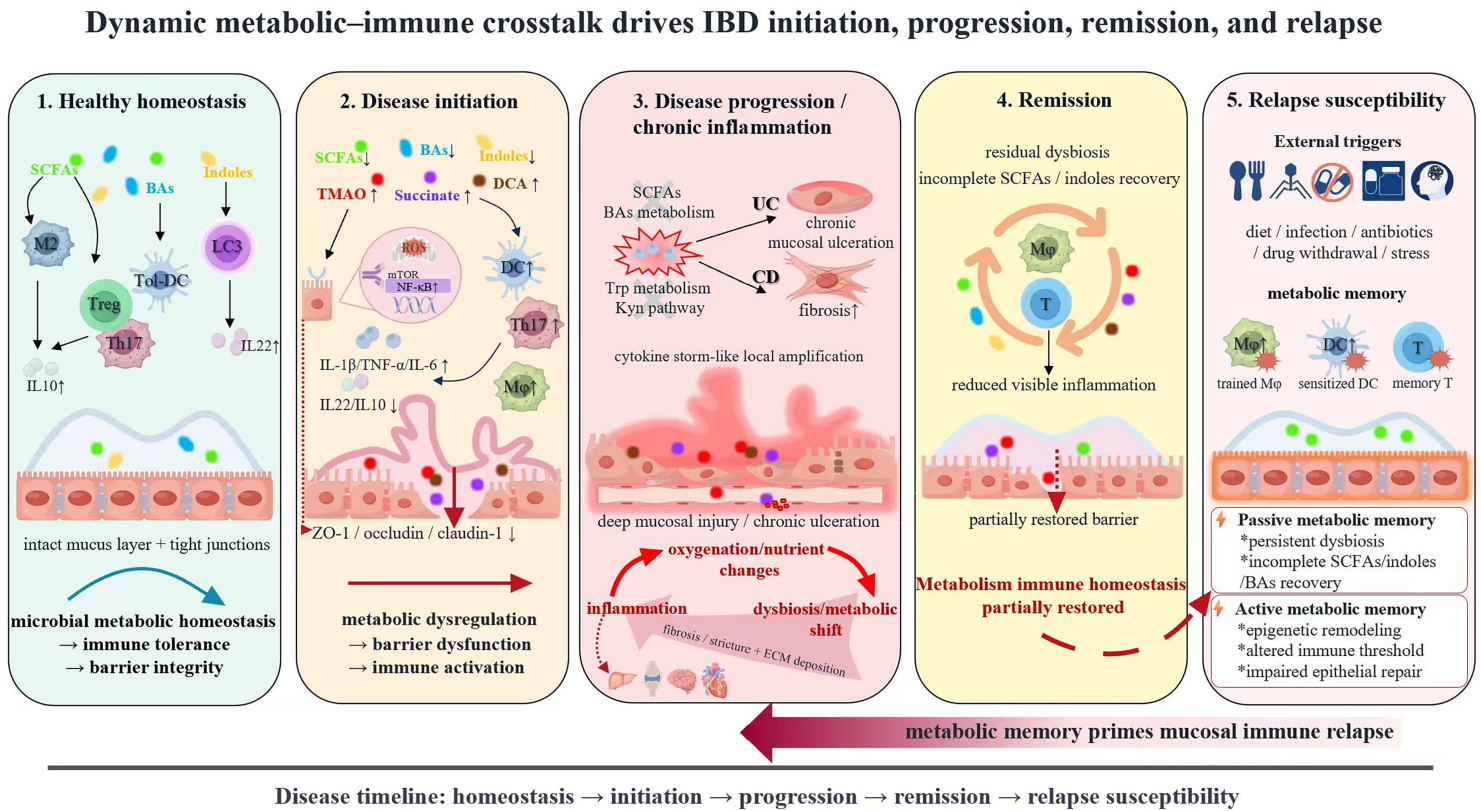
Dynamic interactions between microbial metabolites and mucosal immunity during IBD initiation, progression, and relapse. During IBD initiation, loss of anti-inflammatory metabolites and accumulation of pro-inflammatory metabolites disrupt immune tolerance and epithelial barrier integrity, leading to mucosal immune activation. During disease progression, persistent metabolite–immune feedback amplifies chronic inflammation, tissue remodeling, fibrosis, and extraintestinal manifestations. Even during remission, incomplete restoration of microbial metabolism and residual immune activation may establish metabolic memory and increase relapse susceptibility. The dominant metabolite–immune interactions exhibit stage-specific characteristics throughout disease progression.

Inflammatory bowel disease development represents a dynamic transition from disruption of metabolic–immune homeostasis toward persistent inflammatory imbalance. Under genetically susceptible conditions, environmental exposures may induce alterations in microbial metabolic profiles, accompanied by reduced availability of immunoregulatory metabolites and increased inflammatory-associated metabolic signals. These changes may participate in the initiation, amplification, and persistence of mucosal inflammation. Importantly, the functional role of metabolic–immune interactions is not fixed but varies across different disease stages, reflecting stage-specific remodeling of microbial ecology, metabolite availability, and host immune responses. The dynamic interactions between microbial metabolites and mucosal immunity during IBD progression are illustrated in Figure 3.

It should be noted that Crohn’s disease (CD) and ulcerative colitis (UC) exhibit substantial heterogeneity in microbial composition, metabolic signatures, immune profiles, and therapeutic responses. CD is typically characterized by transmural inflammation and stronger Th1/Th17-associated immune activation, whereas UC primarily involves mucosal inflammation with distinct immune characteristics. Correspondingly, differences in microbial ecological structures and metabolite alterations may contribute to their divergent pathological trajectories. Therefore, although many metabolic–immune mechanisms are shared across IBD subtypes, their relative contribution may vary between CD and UC.

### 4.1 Metabolic–immune imbalance during IBD initiation

The initiation stage of IBD may represent an early window during which intestinal metabolic–immune homeostasis becomes disrupted. During this phase, alterations in microbial metabolic profiles may precede overt pathological changes and clinical manifestations. A characteristic feature is the coexistence of reduced immunoregulatory metabolites and increased inflammatory-associated metabolic signals, which may contribute to the transition from immune tolerance toward a more activated mucosal immune state (Lemser et al., 2026). Importantly, this process is more likely to reflect a global disturbance of the metabolic–immune network rather than inflammation initiation driven by a single metabolite.

At this stage, alterations in SCFA production, secondary bile acid metabolism, and tryptophan-derived metabolites may collectively influence immune tolerance-associated pathways. Reduced butyrate availability, for example, has been associated with impaired regulation of innate immune homeostasis. Through HDAC-related mechanisms and GPR109A signaling, reduced butyrate signaling may be linked to altered macrophage functional polarization, dendritic cell activation status, and Treg maintenance capacity (Allwang et al., 2026; Woo and Alenghat, 2022; Zhang Y. et al., 2024). However, these observations mainly originate from mechanistic and experimental studies, and the precise causal contribution of early metabolic alterations to human IBD initiation requires further longitudinal validation.

Altered secondary bile acid metabolism may also be associated with immune imbalance during early disease development. Reduced levels of specific immunoregulatory bile acid derivatives, such as 3β-hydroxydeoxycholic acid and isoallolithocholic acid, have been linked to altered dendritic cell activation and Th17-associated immune responses through bile acid receptor-related pathways (Hu et al., 2022). Similarly, disruption of tryptophan metabolism may influence mucosal immune regulation through reduced AHR signaling activity, potentially affecting ILC3-derived IL-22 responses and epithelial defense mechanisms (Wang et al., 2023). Together, these metabolic alterations may contribute to a less stable immune tolerance environment.

Meanwhile, alterations in branched-chain and aromatic amino acid metabolism, increased inflammatory-associated metabolites such as TMAO, and disturbed bile acid homeostasis may be associated with impaired epithelial barrier function and oxidative stress responses (Niu et al., 2025). These changes may increase microbial–host interaction opportunities and further reinforce local inflammatory susceptibility.

Beyond molecular alterations, spatial differences in metabolite distribution along the intestinal tract may contribute to regional vulnerability during IBD development (Lyras et al., 2026). Under physiological conditions, the distal ileum and colon exhibit high microbial fermentation activity due to efficient bile acid transformation, abundant fermentable substrates, and relatively stable anaerobic conditions, resulting in enrichment of microbial fermentation products including SCFAs and branched-chain fatty acids (Li C. et al., 2026; van Deuren et al., 2025). However, inflammatory conditions may disrupt these physiological metabolic gradients and alter the relationship between metabolite exposure and mucosal immune regulation (Mann et al., 2024).

The distal colon, which contains the highest microbial density and fermentation activity, is exposed to relatively high levels of microbial metabolites, creating a unique metabolic environment for epithelial and immune cells. Under healthy conditions, this environment supports barrier maintenance and immune regulation. However, in the context of dysbiosis and barrier impairment, altered metabolite composition and abnormal microbial distribution may modify local immune thresholds and inflammatory susceptibility. For example, depletion of SCFA-producing bacteria together with expansion of facultative anaerobic microorganisms may contribute to changes in local redox conditions and metabolic outputs, potentially influencing regional inflammatory patterns (Gong et al., 2026; Wang and Mackay, 2024; Xu et al., 2026).

### 4.2 Metabolite-mediated inflammatory amplification and chronic remodeling during IBD progression

As IBD progresses, early metabolic disturbances may become integrated with persistent immune activation and tissue remodeling processes. During this stage, metabolic–immune interactions may contribute to sustained inflammatory activity through continuous alteration of epithelial repair capacity, immune cell function, and tissue homeostasis (Zhang et al., 2026).

Persistent reduction of immunoregulatory metabolic signals may impair epithelial energy metabolism, autophagy regulation, and repair responses, whereas accumulation of inflammation-associated metabolites may maintain activation of pathways including NF-κB, HIF-1α, and TGF-β/SMAD signaling (Bao et al., 2025; Bhagavatula et al., 2025). The long-term interaction between metabolic imbalance and immune activation may contribute to a pathological state characterized by persistent tissue injury and incomplete repair.

At the structural level, epithelial damage, extracellular matrix deposition, and fibroblast activation collectively participate in intestinal remodeling. These processes exhibit distinct manifestations between IBD subtypes, with CD more frequently characterized by transmural inflammation and intestinal stricture formation, whereas UC is typically associated with chronic mucosal inflammation and impaired epithelial regeneration (Morey et al., 2026; Vieujean et al., 2021).

Beyond the intestinal compartment, microbial-derived metabolites entering the circulation may influence extraintestinal manifestations by modulating endothelial function and systemic immune activation. Circulating inflammatory-associated metabolites, including lipopolysaccharide, trimethylamine N-oxide (TMAO), and altered bile acid derivatives, have been associated with endothelial NF-κB activation, increased adhesion molecule expression, and enhanced immune cell recruitment (Huang et al., 2024; Witkowski et al., 2022). Although these metabolites have been linked to cardiovascular, hepatobiliary, and neurological complications, the precise causal relationships between circulating metabolites and extraintestinal manifestations require further validation.

Importantly, the effects of circulating metabolites may not be restricted to acute inflammatory responses. Persistent exposure to abnormal metabolic signals may induce long-term functional alterations in endothelial cells and immune cells through sustained signaling activation and epigenetic remodeling. These processes may generate a persistent systemic susceptibility state resembling a “systemic metabolic memory,” potentially explaining why certain extraintestinal manifestations may persist or recur despite improvement of intestinal inflammation.

### 4.3 Metabolic memory and inflammatory relapse during remission

The chronic relapsing nature of IBD suggests that clinical remission does not necessarily indicate complete restoration of metabolic–immune homeostasis. In some patients, residual alterations in microbial composition, metabolite profiles, and immune function may persist despite substantial reduction of inflammatory activity (Braun et al., 2026). This incomplete restoration of metabolic regulation, characterized by insufficient recovery of immunoregulatory metabolites and persistent low-grade inflammatory signals, may represent an important basis underlying the concept of microbial metabolic memory. Alterations in SCFA availability, tryptophan-derived metabolites, and bile acid metabolism may contribute to impaired immune tolerance and unstable epithelial barrier maintenance during remission (Peng et al., 2024).

Importantly, this metabolic vulnerability may not simply represent a passive residual abnormality but could be maintained through continuous interactions between microbial ecology, metabolite availability, and host immune responses. Metabolic disturbances may influence epigenetic regulation, immune response thresholds, and epithelial repair capacity, thereby increasing susceptibility to inflammatory reactivation upon subsequent environmental stimulation (Yuan et al., 2025). Meanwhile, incomplete ecological restoration of the microbiota may further restrict recovery of metabolic homeostasis, allowing this vulnerable state to persist over time (Pisani et al., 2022).

A potential self-maintaining inflammatory–metabolic feedback loop may further explain this process. Residual low-grade inflammation during remission may induce subtle alterations in intestinal redox conditions and increase luminal oxygen availability, thereby limiting the recovery of strictly anaerobic butyrate-producing bacteria, including *Faecalibacterium* and *Roseburia* species (Lee et al., 2024). Reduced butyrate availability may subsequently weaken HDAC inhibition-mediated regulation of Foxp3-associated chromatin accessibility, thereby affecting Treg stability and restoration of immune tolerance (McBride et al., 2023). Reduced regulatory immune capacity and lowered inflammatory thresholds may in turn maintain a low-grade inflammatory environment, further reinforcing oxidative conditions and limiting recovery of butyrate-producing microbial communities (Gong et al., 2025). Collectively, this potential feedback loop of “low-grade inflammation–increased oxygen availability–loss of butyrate-producing bacteria–reduced butyrate production–impaired Treg maintenance–persistent inflammation” provides a mechanistic framework for understanding relapse susceptibility during remission.

However, evidence supporting microbial metabolic memory as a determinant of IBD recurrence remains emerging. Current studies suggest that fecal or circulating metabolite profiles during remission may be associated with subsequent disease activity, relapse risk, or treatment responses (Levhar et al., 2025). Nevertheless, differences in analytical platforms, disease subtypes, medication exposure, and follow-up designs limit direct causal interpretation. Therefore, microbial metabolic memory should currently be considered an emerging explanatory framework integrating residual metabolic abnormalities and immune susceptibility rather than an established independent pathogenic mechanism.

Furthermore, CD and UC may exhibit distinct metabolic remodeling patterns, which could contribute to their divergent inflammatory phenotypes and clinical trajectories (Radhakrishnan et al., 2025). Previous studies have suggested that alterations in tryptophan metabolism and kynurenine pathway activity may be relatively prominent in CD, whereas reduced SCFA availability and disturbed bile acid metabolism may represent more characteristic features reported in UC (Wu J. et al., 2024). However, these differences remain incompletely defined and require validation through larger longitudinal multi-omics studies. Understanding subtype-specific metabolic signatures may provide additional insights into disease heterogeneity and support future precision therapeutic strategies.

## 5 Therapeutic strategies and translational prospects targeting microbial Metabolite–Mucosal immune crosstalk

Given the important role of microbial metabolites as potential mediators linking gut microbiota alterations with mucosal immune dysfunction in IBD, targeting metabolite–immune interactions has emerged as a promising therapeutic direction. However, it should be noted that current evidence supporting these approaches remains highly heterogeneous, ranging from randomized controlled trials (RCTs) and small-scale exploratory clinical studies to preclinical investigations. These strategies therefore differ substantially in terms of evidence strength, translational maturity, and clinical applicability.

**FIGURE 4:**
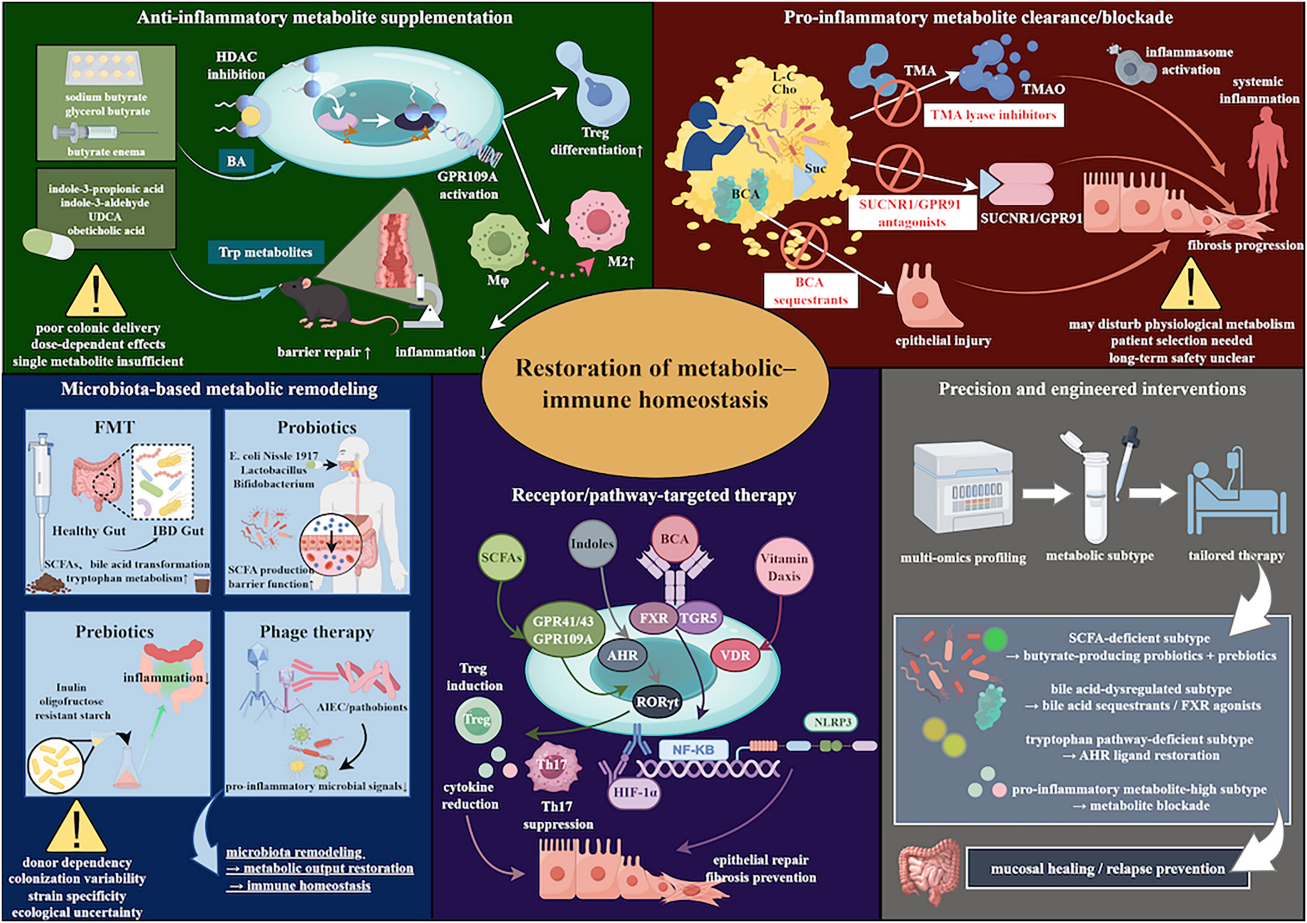
Therapeutic strategies targeting microbial metabolite–mucosal immune crosstalk in IBD. Emerging therapeutic strategies targeting metabolite–immune crosstalk include supplementation of anti-inflammatory metabolites, inhibition of pro-inflammatory metabolic signaling, microbiota-directed metabolic remodeling, and precision metabolite-based interventions. Current approaches involve short-chain fatty acid supplementation, bile acid modulation, engineered probiotics, fecal microbiota transplantation, phage therapy, receptor-targeted drugs, and metabolomics-guided precision treatment. These strategies aim to restore intestinal metabolic–immune homeostasis and improve long-term disease control in IBD.

Furthermore, clinical responses may vary between ulcerative colitis (UC) and Crohn’s disease (CD), given their distinct inflammatory distributions, immune characteristics, and patterns of epithelial barrier disruption. Current therapeutic approaches targeting microbial metabolite–immune interactions mainly include direct supplementation of immunoregulatory metabolites, elimination or blockade of pro-inflammatory metabolic signals, metabolite receptor-targeted interventions, and microbiota-mediated reconstruction of metabolic pathways (Figure 4).

### 5.1 Direct supplementation of immunoregulatory metabolites

Direct supplementation of immunoregulatory metabolites represents one of the most straightforward strategies for restoring metabolic–immune balance. This approach aims to replenish beneficial microbial metabolites, including short-chain fatty acids (SCFAs), tryptophan derivatives, and secondary bile acids, thereby potentially restoring local immune regulation and attenuating excessive inflammatory responses (Xu et al., 2025).

Among these strategies, SCFA supplementation, particularly butyrate replacement, has been extensively investigated. Oral administration of sodium butyrate, tributyrin, or rectal butyrate enemas may increase local butyrate availability and regulate mucosal immune responses through mechanisms involving histone deacetylase (HDAC) inhibition and GPR109A signaling. These effects may promote regulatory T cell (Treg) differentiation, support macrophage anti-inflammatory polarization, and enhance epithelial barrier integrity (Tang et al., 2025).

Clinical studies have suggested that butyrate enemas may improve clinical symptoms and endoscopic inflammatory activity in some patients with mild-to-moderate UC, with generally favorable safety profiles (Hodgkinson et al., 2023). However, current clinical evidence remains relatively limited, and most studies involve small patient populations. In addition, microbial-derived metabolites such as indole-3-propionic acid and indole-3-aldehyde, as well as secondary bile acids including ursodeoxycholic acid and obeticholic acid, have demonstrated anti-inflammatory effects mainly in experimental models. Their therapeutic efficacy and safety in human IBD remain under investigation (Li et al., 2025; Wang G. et al., 2024).

Despite its strong mechanistic rationale, direct metabolite supplementation faces several translational challenges. First, exogenously administered metabolites often exhibit limited stability and bioavailability within the gastrointestinal tract due to absorption or degradation before reaching the colon, thereby restricting effective colon-targeted delivery (Guo et al., 2026). Second, supplementation of individual metabolites may not be sufficient to restore the complexity of the endogenous metabolic network, which may limit therapeutic efficacy. Moreover, the biological effects of metabolites are highly dependent on dosage, cellular context, and inflammatory status, with the possibility of distinct or even opposing effects under different microenvironmental conditions (Sun et al., 2025). Future studies should therefore focus on improving colon-specific delivery systems, evaluating combined metabolite supplementation strategies, and developing patient stratification approaches to enhance translational potential.

### 5.2 Elimination of pro-inflammatory metabolites and metabolic signal blockade

In contrast to metabolite replacement strategies, another therapeutic approach aims to reduce the accumulation or biological activity of inflammation-associated metabolites by limiting their production, inhibiting related metabolic enzymes, or blocking downstream receptor signaling. These approaches may help attenuate persistent inflammatory signaling and prevent excessive amplification of immune responses (Li A. et al., 2026).

Trimethylamine N-oxide (TMAO) represents one potential target in this category. TMAO production depends on microbial conversion of dietary nutrients such as choline and L-carnitine into trimethylamine, followed by hepatic oxidation. Pharmacological inhibition of microbial trimethylamine lyase activity may reduce TMAO generation and circulating TMAO levels (Benson et al., 2023). Experimental studies have suggested that this strategy may alleviate TMAO-associated intestinal barrier impairment and systemic inflammatory responses, although its therapeutic relevance in human IBD remains to be established (Chen J. et al., 2025; Wang T. et al., 2026).

Similarly, antagonizing succinate receptor GPR91 (also known as SUCNR1) has been proposed as a potential strategy to limit succinate-driven inflammatory signaling and fibroblast activation. Preclinical models suggest that GPR91 inhibition may reduce fibrosis-related responses, providing a possible therapeutic avenue for intestinal fibrotic complications, particularly in CD; however, clinical validation remains lacking (Xie L. et al., 2026).

For cytotoxic or inflammation-associated bile acid metabolites, such as deoxycholic acid, bile acid sequestrants may reduce intestinal exposure by binding these compounds and promoting fecal elimination. Clinical observations suggest that bile acid sequestrants may improve diarrhea symptoms and intestinal discomfort in selected IBD patients, particularly those with bile acid malabsorption (Borup et al., 2023). Nevertheless, excessive interference with enterohepatic bile acid circulation may affect physiological bile acid functions and potentially impair absorption of fat-soluble vitamins. Therefore, careful patient selection and long-term safety evaluation are required before broader clinical application.

### 5.3 Microbiota-based reconstruction of metabolic pathways

Modulating gut microbial composition and function to restore metabolic pathway activity represents a potential strategy for correcting metabolic abnormalities at their ecological origin. Unlike approaches that simply aim to alter microbial abundance, microbiota-based interventions within the framework of this review emphasize the restoration of functional metabolic capacity, including SCFA production, bile acid transformation, tryptophan metabolism, and other immune-related metabolic pathways, thereby potentially re-establishing metabolic–immune homeostasis.

Current strategies mainly include fecal microbiota transplantation (FMT), probiotics, prebiotics, and phage-based therapies, with their mechanisms, clinical evidence, and limitations summarized in Table 2.

**TABLE 2:** Microbiota-mediated metabolic remodeling strategies in IBD.

Strategy	Mechanism	Evidence	Limitations	Ref.
FMT	Microbiota reconstruction; SCFA, bile acid and tryptophan pathway restoration	Clinical/RCT evidence mainly in UC	Donor dependence; variable efficacy; standardization and safety concerns	Gogokhia et al., 2025
Probiotics	Functional strain supplementation; SCFA production; barrier and Treg modulation	Clinical evidence mainly for UC maintenance/adjunctive therapy	Limited colonization; strain specificity; weak CD evidence	Yu P. et al., 2026
Prebiotics	Fermentable substrates; enrichment of SCFA-producing bacteria	Small clinical/exploratory studies	GI discomfort; baseline microbiota dependence; caution in stricturing disease	Correa et al., 2024; Medawar et al., 2024
Phage therapy	Targeted depletion of pathobionts such as AIEC	Preclinical/early exploratory stage	Narrow host range; resistance; delivery, ecological and regulatory challenges	Federici et al., 2022; Titécat et al., 2022

Fecal microbiota transplantation involves the transfer of a complete microbial ecosystem from healthy donors and may partially restore microbial diversity and metabolic functionality. Several clinical studies have suggested that FMT may induce clinical remission or endoscopic improvement in a subset of patients with UC, although treatment responses remain highly heterogeneous. Current limitations include donor dependency, insufficient standardization, potential pathogen transmission risks, and uncertainty regarding long-term ecological stability. In contrast, evidence supporting FMT in CD remains relatively limited.

Probiotics may contribute to metabolic restoration by transient intestinal colonization or fermentation of dietary substrates, thereby enhancing SCFA production and modulating immune responses, including Treg-associated regulation and epithelial barrier maintenance. Some specific strains have shown potential benefits in UC maintenance therapy; however, their efficacy remains strain-specific, intestinal persistence is often limited, and consistent clinical benefits in CD have not been established.

Prebiotics aim to indirectly improve metabolic profiles by providing fermentable substrates that promote beneficial microbial communities and enhance microbial metabolite production. Although some studies suggest potential improvements in microbial composition and inflammatory parameters, their effects are strongly influenced by baseline microbiota characteristics. In addition, careful consideration is required in patients with intestinal strictures or obstruction risks.

Phage therapy represents a more precise microbiota-targeted approach by selectively eliminating pathogenic or inflammation-associated bacterial populations and reducing related inflammatory stimuli or abnormal metabolite production. However, current applications remain largely at the preclinical or early clinical stage. Major translational challenges include limited host range, emergence of bacterial resistance or escape mechanisms, insufficient intestinal delivery efficiency, uncertain ecological consequences, and the lack of standardized manufacturing and regulatory frameworks.

Overall, FMT represents a strategy for broad ecological reconstruction, whereas probiotics and prebiotics may be more suitable for long-term modulation of microbial metabolic functions. Phage-based approaches provide a higher degree of microbial targeting precision but remain under early development. Importantly, all these strategies are affected by substantial inter-individual variability in microbiota composition, disease subtype, and treatment background, resulting in heterogeneous clinical outcomes. Future microbiota-based interventions should therefore shift from simple microbial restructuring toward restoration of functional metabolic networks and integration with approaches aimed at rebuilding mucosal immune homeostasis.

### 5.4 Emerging precision intervention strategies

With advances in precision medicine, individualized therapeutic strategies based on patient-specific metabolic characteristics have become an emerging research direction. Integrating metabolomic profiling of intestinal and circulating metabolites may enable identification of distinct metabolic abnormalities and facilitate personalized intervention strategies.

For example, patients characterized by reduced SCFA availability may potentially benefit from approaches combining butyrate-producing probiotics with appropriate prebiotic substrates, whereas individuals with disturbed bile acid metabolism may represent candidates for bile acid-targeted interventions, including bile acid sequestrants or FXR agonist-based strategies (Zhang M. et al., 2024). However, these approaches remain largely exploratory and require validation in appropriately designed clinical studies.

In addition, engineered microbial systems represent an emerging strategy for precise metabolic regulation. Genetically modified probiotics may be designed to sense inflammatory microenvironments and locally produce anti-inflammatory metabolites or metabolic enzymes, thereby enabling targeted modulation of intestinal metabolic signals while minimizing systemic exposure (Ye et al., 2026). Nevertheless, several challenges remain, including microbial colonization efficiency, host immune responses, biosafety concerns, and regulatory requirements.

To establish a clearer connection between therapeutic strategies and disease progression, this review further incorporates a disease-stage perspective into the existing intervention-based classification framework. From a pathological perspective, different stages of IBD are characterized by distinct metabolic–immune abnormalities, and therapeutic strategies may therefore exert their greatest potential at different time windows. Specifically, direct supplementation of immunoregulatory metabolites may primarily target the initiation stage by restoring metabolic balance and limiting early inflammatory activation; blockade of inflammation-associated metabolic signals may be more relevant during disease progression by reducing inflammatory amplification and tissue damage; whereas microbiota-mediated metabolic reconstruction and precision interventions may contribute to restoration of metabolic networks during remission and potentially reduce relapse susceptibility associated with persistent metabolic abnormalities.

This disease-stage perspective provides an additional conceptual framework without altering the existing classification of therapeutic approaches and helps establish a more coherent relationship between metabolic abnormalities, disease evolution, and targeted interventions.

Overall, therapeutic strategies targeting microbial metabolite–mucosal immune interactions are gradually evolving from single-factor modulation toward integrated regulation of metabolic networks. Different disease stages may involve distinct metabolic abnormalities and corresponding therapeutic opportunities. However, current approaches still lack a unified mechanistic framework linking metabolic signatures with treatment selection. Future studies should integrate longitudinal multi-omics profiling, metabolic stratification, and mechanism-driven clinical trials to facilitate the transition from empirical intervention toward precision therapy.

## 6 Future perspectives

The bidirectional interaction between microbial metabolites and mucosal immunity represents an important mechanistic link connecting gut microbial dysfunction with immune-mediated inflammation in IBD. Microbial-derived metabolites, including SCFAs, tryptophan derivatives, bile acids, polyamines, and other bioactive molecules, may regulate intestinal homeostasis through modulation of epithelial barrier integrity, innate immune responses, adaptive immune differentiation, and inflammatory thresholds. In IBD, reduced availability of immunoregulatory metabolites, accumulation of inflammation-associated metabolic signals, and abnormal immune activation may interact with each other, potentially contributing to barrier disruption, persistent inflammation, tissue injury, and relapse susceptibility. Meanwhile, mucosal immune responses may reciprocally reshape microbial composition and metabolic outputs through cytokine networks, antimicrobial peptides, IgA responses, oxygen availability, and ecological niche alterations, suggesting that metabolic abnormalities may represent not merely consequences of dysbiosis but also potential contributors to chronic disease persistence.

Despite the promising potential of microbial metabolite–immune interactions as therapeutic targets, substantial challenges remain before clinical translation can be achieved. First, many microbial metabolites exhibit limited stability and bioavailability within the gastrointestinal tract, making effective colon-targeted delivery and maintenance of local concentrations difficult. Second, current experimental models often differ from human IBD in microbial composition, metabolic pathways, and immune responses, limiting direct extrapolation of mechanistic findings. Third, substantial inter-individual variability in baseline metabolomic profiles, together with differences in disease subtype, medication exposure, and dietary background, complicates the establishment of standardized clinical endpoints and therapeutic response criteria.

Future research should therefore focus on improving metabolite delivery systems, validating mechanisms across species, and developing metabolomic-based patient stratification strategies. In addition, standardized metabolite measurement platforms and prospective clinical trials involving biologically defined patient subgroups will be essential for bridging mechanistic discoveries and clinical applications. Through these efforts, the field may progress from descriptive characterization of microbial metabolite alterations toward mechanism-guided and precision metabolic interventions in IBD.
